# Perceived discrimination as a predictor of cyberbullying: the mediating role of self-esteem and moderating role of self-compassion

**DOI:** 10.3389/fpsyg.2024.1499759

**Published:** 2024-12-24

**Authors:** Qianfeng Li, Sicheng Shang, Jiawen Du, Jia Wu, Shaobei Xiao

**Affiliations:** ^1^School of Psychology, Hainan Normal University, Haikou, China; ^2^Adolescent Psychological Development and Education Center of Hainan, Hainan Normal University, Haikou, China; ^3^School of Elementary Education, Hainan Normal University, Haikou, China; ^4^Student Affairs Office, Guangzhou City University of Technology, Guangzhou, China

**Keywords:** perceived discrimination, cyberbullying, self-esteem, self-compassion, compassionate self-responding, reduced uncompassionate self-responding, longitudinal study

## Abstract

With the popularity of the internet, cyberbullying has emerged as an increasingly serious social issue, particularly affecting college students’ behavioral health. This study explores the relationship between perceived discrimination and cyberbullying, as well as the mediating role of self-esteem and the moderating role of self-compassion (SC) in this relationship. Using a longitudinal design, data were collected from 892 Chinese college students (414 females, 478 males) in two waves spanning 1 year. The present study measured the compassionate self-responding (CS) and reduced uncompassionate self-responding (RUS) as the two dimensions of self-compassion. The study found that (1) perceived discrimination was significantly and positively correlated with cyberbullying (*r* = 0.085, *p* < 0.05); (2) self-esteem mediated the relationship between perceived discrimination and cyberbullying (Indirect effect = 0.010, SE = 0.004, Boot 95% CI [0.003, 0.019]); (3) RUS moderated both the direct relationship between perceived discrimination and cyberbullying (*b* = −0.045, SE = 0.019, 95% CI [−0.082, −0.008]) as well as the indirect relationship through self-esteem (*b* = −0.081, SE = 0.033, 95% CI [−0.146, −0.015]). Simple slope analysis revealed that college students with high RUS exhibited less cyberbullying when facing discrimination, while those low in RUS were more likely to engage in cyberbullying. The SC exhibited similar moderating effects, but CS did not demonstrate significant moderating effects in those relationships. This study sheds light on the complex interplay between perceived discrimination, self-esteem, self-compassion, and cyberbullying and offers novel insights into the psychological mechanisms underlying cyberbullying among college students. The findings underscore the importance of interventions aimed at enhancing self-esteem and fostering self-compassion, particularly by addressing uncompassionate self-responding, as a strategy to prevent cyberbullying among disadvantaged college students.

## Introduction

With the continuous rise in internet penetration, people enjoy the convenience of online social interactions and also face the potential threat of cyberbullying. Cyberbullying can lead to a range of physical and mental health problems in victims, such as depression, suicidality, and anti-social behavior (Bansal et al., 2023; Wolke and Lereya, 2015). Cyberbullying refers to an aggressive, intentional act carried out by a group or individual, using electronic forms of contact, repeatedly and over time against a victim who cannot easily defend him or herself (Smith et al., 2008). College students’ lives are highly dependent on electronic devices, and using online social networks has become one of their primary methods of socializing. This dependency increases their susceptibility to becoming victims of the harms associated with virtual, anonymous, and instantaneous cyberbullying through social media, or even potential perpetrators (Shaikh et al., 2021). Although numerous studies have explored the negative effects of cyberbullying on the global health and well-being of victims, there has been limited research into potential predictors of why and when young people are more likely to become involved in cyberbullying. This research explored how social class-based discrimination links to cyberbullying among college students over time, providing critical insights for developing interventions to reduce cyberbullying behaviors among youth experiencing discrimination.

### Perceived discrimination among college students

It is essential to understand the role of specific social circumstances in cyberbullying among college students. Studies on the predictive factors of cyberbullying have shown that socioeconomic status, peer norms, and parental educational levels are significant influences (Bullo and Schulz, 2022; Lapierre and Dane, 2020; Uludasdemir and Kucuk, 2019). However, research is lacking on the risk factors of social circumstances related to cyberbullying behavior among college students (Peterson and Densley, 2017; Shaikh et al., 2021). A considerable number of young people are likely to face significant social challenges, such as perceived discrimination, due to being in the transitional phase of their social roles (Wu et al., 2022). For example, the rising cost of higher education is forcing many young people to rely on student loans to cover tuition fees and increasing their financial burden (Avery and Turner, 2012; Britt et al., 2017; Xiao et al., 2020). Meanwhile, as socioeconomic inequalities and structural barriers impede social mobility, the job market is becoming increasingly competitive, making it difficult for many students to secure satisfactory employment (Changjun and Liping, 2019; Chu et al., 2020). These factors contribute to a perception of social class discrimination among young people and lead them to a sense of greater inequality and differential treatment in their lives and career development. Facing severe social challenges, young people are more likely to use social networks covertly to express their dissatisfaction and compensate for what they feel is lacking in their offline social activities.

Frustration-aggression hypothesis (Berkowitz, 1989) provides theoretical support to understand the relationship between perceived discrimination and cyberbullying. Berkowitz (1989) argued that frustration can generate a tendency towards aggressive behavior because it instils aversive feelings and restricts people from achieving their goals. Even in situations not involving direct and personal discrimination, such as perceived disadvantages associated with socioeconomic status or race, people may still exhibit aggressive behavior. These arguments are supported by numerous empirical findings that perceived racial discrimination is positively related to aggressive behavior among youth (Burt et al., 2012; Hautala and Sittner, 2019). An empirical study by Bedrosova et al. (2022) demonstrated that both individual-based discrimination (e.g., related to physical characteristics such as height or weight) and group-based discrimination (e.g., linked to family origin) were significantly associated with increased cyberbullying behaviors among adolescents. Moreover, a longitudinal study indicated that socioeconomic status disadvantage can predict higher aggressive behavior 1 year later (Santiago et al., 2011). However, to the best of our knowledge, there is no direct empirical evidence for a longitudinal relationship between perceived discrimination and cyberbullying. To address this gap in the literature, the current study explores the association between perceived discrimination and cyberbullying, proposing the following hypothesis:

*Hypothesis 1: Perceived discrimination is significantly associated with higher rates of cyberbullying among students who experience social discrimination.*

### Roles of self-esteem and self-compassion

Studies have indicated that frustration, such as that stemming from discriminatory experience, can produce compliance with social norms rather than aggression (Leander et al., 2020), and others have suggested that the mechanism of the relationship between frustration and aggression needs further exploration (Berkowitz, 1989; Hautala and Sittner, 2019). The self-evaluation maintenance theory suggests that individuals strived to maintain a positive self-concept, and when their self-image was threatened (e.g., by perceived discrimination), they may resort to externalizing behaviors to restore their sense of self-worth (Tesser, 1988). As a global evaluation of a person’s own value and capacity, self-esteem may serve as a potential pathway to understand the complex relationship between perceived discrimination and cyberbullying. Low self-esteem is one of the outcomes most commonly associated with discrimination (Alsawalqa, 2021; Zhang et al., 2023). Moreover, Baumeister et al. (1996) suggested that aggression occurs when an individuals’ self-evaluation is threatened by negative evaluations from others, such as through discrimination. Self-esteem reflects an individual’s subjective assessment of their important social relationships. Respect and a sense of belonging from others were fundamental psychological needs (Kruglanski et al., 2023; Liu et al., 2024). When these needs are frustrated, particularly by external rejection or insult, the individual’s self-esteem was threatened, triggering feelings of anger and hostility towards others. Thus, individuals perceive a threat to their self-esteem, they may adopt defensive or aggressive behaviors to protect and recover it.

Research has indicated that perceived discrimination can damage self-esteem and then increase online bullying behavior (Lei et al., 2020). However, the relationship between self-esteem and cyberbullying is complex (Shi et al., 2017). On the one hand, studies have indicated a negative correlation between self-esteem and cyberbullying (Ding et al., 2018). On the other hand, other researchers contend that this correlation is positive, suggesting that individuals with higher self-esteem are more likely to engage in cyberbullying (Fan et al., 2019). As a trait-like individual characteristic, self-esteem comprises both stable and unstable components (Braun et al., 2021). This implies that the relationship between self-esteem and cyberbullying may be affected by other factors (Lei et al., 2020). Thus, the present study further explores the role of self-esteem in the relationship between perceived discrimination and cyberbullying, specifically by examining when threats to self-esteem become linked to cyberbullying. We propose the following hypothesis:

*Hypothesis 2: The positive effect of perceived discrimination on cyberbullying is mediated by self-esteem.*

Self-compassion has been found to weaken the association between a frustrating situation and cyberbullying perpetration (Geng and Lei, 2021). Self-compassion, defined as compassion for one’s own suffering, is an effective approach to managing distressing thoughts and emotions and can promote mental and physical well-being (Neff, 2023). According to stress coping theory, individuals adopt different coping styles when confronted with stressful situations (Lazarus, 1993). As a positive coping strategy, self-compassion can mitigate aggression resulting from discrimination. College students can experience self-indifference and self-doubt due to unfair treatment (Wondra and McCrea, 2022), but self-compassion can help mitigate the negative effect of discriminatory experiences and protect their emotional well-being in tough social situations (Li et al., 2022). Accordingly, when the degree of negative emotion stimulated by a frustrating experience (e.g., discrimination) is reduced, an individual’s tendency towards aggression may also weaken. Furthermore, the association between damaged self-esteem and cyberbullying can also be moderated by self-compassion. Recent empirical studies have shown that self-compassion can decrease cyberbullying perpetration among college students and enhance the psychological functioning of young people with low self-esteem who encounter stigma or discrimination (Langford et al., 2022). In line with these previous findings, we propose that self-compassion buffers the direct association between perceived discrimination and cyberbullying and moderates the indirect association between perceived discrimination and cyberbullying through self-esteem.

*Hypothesis 3: The positive relationship between perceived discrimination and cyberbullying is weaker among college students with high levels of self-compassion than among those with low levels of self-compassion.*

*Hypothesis 4: The positive effect of perceived discrimination on cyberbullying through low self-esteem is weaker among college students with high levels of self-compassion than among those with low levels of self-compassion.*

Furthermore, the moderating role of different components of self-compassion remains underexplored in the literature. Recent studies have introduced a new framework that divides self-compassion into two components that distinguish the positive and reduced negative aspects of self-compassion: compassionate self-response (CS) (self-kindness, common humanity, mindfulness) and reducing uncompassionate responses (RUS) (self-judgment, isolation, over-identification) (Neff et al., 2018). Although CS and RUS have been shown to alleviate mental health problems and enhance healthy psychological functioning (Germer and Neff, 2013; Raes, 2011), RUS is more strongly linked to self-evaluation than CS (Neff et al., 2018). Individuals with low self-esteem often struggled with social connections, leading to poor social adaptation and increased aggression (Hirschi, 1969). Uncompassionate self-responding, characterized by dehumanization, allowed individuals to minimize the severity of their bullying behavior and avoid self-sanctions, increasing the likelihood of online aggression (Runions et al., 2019). Reducing uncompassionate self-responding is crucial for mitigating the impact of low self-esteem on online bullying.

**Figure 1:**
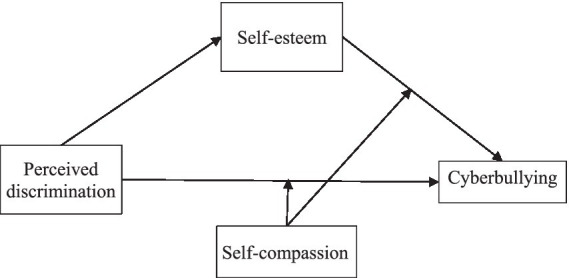
The proposed model.

Reducing uncompassionate responses is more likely to help break the vicious cycle of negative self-evaluation and self-criticism, which in turn deceases an individual’s aversive feelings and consequently reduces cyberbullying (Ding et al., 2018). An empirical study showed that CS and RUS differed in their moderating effects on the relationship between perceived discrimination and psychopathological outcomes (Li et al., 2022). To summarize, the present study examines the moderating effects of SC, RUS, and CS on the indirect and direct relationships between perceived discrimination and cyberbullying (the proposed model is shown in the Figure 1).

## Method

### Participants and procedure

We distributed 1,000 questionnaires to eligible participants, and received 909 (90.9%) completed responses at the first survey. During the T2 data collection, 17 students failed to return their answers. Finally, data were collected from 892 university students (414 females and 478 males) across two survey waves with a one-year interval. We recruited eligible participants (full-time undergraduates aged 18 years and over) through the student affairs officers at a university in Guangdong Province, China. We excluded students who were on leave of absence or unable to provide informed consent. These criteria were chosen to ensure the study population represented actively enrolled undergraduates while minimizing potential biases associated with irregular academic status. The average age of the participants at the baseline survey was 20.47 years (SD = 1.37). The educational attainment of 26.7% of the participants’ fathers and 46.9% of their mothers was primary school or below. Their average family monthly income was RMB 6522. The research procedures performed in this study involving human participants were approved by the Institutional Review Board of the XXX University.

Participants were recruited through convenience sampling, with student affairs officers distributing invitations during university events to ensure accessibility. Retention strategies included clear instructions, regular follow-ups, practice credits for academic purposes, and assurances of confidentiality. Data collection occurred in September 2020 (T1) and September 2021 (T2), allowing for a 1-year interval to capture meaningful changes in variables like self-esteem while minimizing bias and fatigue. Data collection was conducted face-to-face during designated university activities for two waves (T1 and T2). To address missing data, we conducted a demographic and baseline analysis comparing participants who completed all three waves with those who dropped out, finding no significant differences. In present study, missing data were handled using full information maximum likelihood (FIML) estimation.

### Measures

The demographic variables (age, gender, parents’ educational level, and household income) were assessed in the baseline survey. Moreover, the subjective socioeconomic status (SSES) of participants was measured by a visual scale with 10-rung ladders (Cheng et al., 2013).

#### Perceived discrimination

Perceived discrimination was assessed using the 6-item Perceived Discrimination Scale (Shen et al., 2009). Participants reported their perceived discrimination on a 5-point Likert scale (ranging from 1 = *strongly disagree* to 5 = *strongly agree*). An example item is “I feel that people treat me differently because of my family social class background.” The mean score of all items was calculated, with a high score representing a higher level of perceived discrimination. In the present study, Cronbach’s *α* of the scale was 0.89.

#### Self-compassion

Participants’ self-compassion was assessed by The Chinese version of the Self-Compassion Scale, with 26 items (Chen et al., 2011; Neff, 2003). The participants responded on their feelings of inadequacy or suffering experience on a 5-point Likert scale (ranging from 1 = *almost never* to 5 = *almost always*). The self-compassion scale consists of six dimensions: self-kindness, common humanity, mindfulness, self-judgement, isolation, and over-identification. The CS score was the mean of self-kindness, common humanity, and mindfulness. The scores for items on the self-judgement, isolation, and over-identification subscales were reverse-coded and their mean scores were calculated as the RUS score. The overall self-compassion (SC) score was taken as the mean score of all items. High RUS, CS, and SC scores indicated higher levels of the corresponding variables. In the present study, Cronbach’s *α* of SC, CS, and RUS was 0.83, 0.87, and 0.86, respectively.

#### Self-esteem

Participants’ self-esteem was measured using the Rosenberg Self-Esteem Scale (RSES; Rosenberg, 1965), a widely validated instrument across cultural contexts, including China. Studies have demonstrated the scale’s reliability and validity in Chinese populations (e.g., Li et al., 2019), with comparable factor structures and internal consistency. In this study, participants rated their agreement with 10 items (e.g., “On the whole, I am satisfied with myself”) on a 4-point Likert scale (1 = strongly disagree to 4 = strongly agree). Five items were reverse-coded, and higher total scores indicated higher self-esteem. Cronbach’s α for this scale in our study was 0.84.

#### Cyberbullying

Cyberbullying was assessed using the Cyber Aggression Scale (Kurek et al., 2019). Participants rated the frequency of specific online behaviors over the past month (e.g., “Posted something online about someone else to make others laugh”) on a 5-point Likert scale (1 = never to 5 = 7 or more times). To ensure its cultural applicability, we conducted a confirmatory factor analysis, which confirmed the one-factor model’s fit (RMSEA = 0.08, CFI = 0.97, TLI = 0.95, SRMR = 0.02). Cronbach’s α for this scale in our study was 0.88, demonstrating strong reliability in the Chinese context.

### Data analyses

Harman’s single factor test was conducted to examine whether common method bias was a serious issue in the present study. The results of an exploratory factor analysis showed that the extracted first component accounted for 23.57% of the total variance, which indicated that the common method bias was not severe.

Descriptive statistics were generated and correlation analyses performed to describe the demographic information of the participants and examine the bivariate correlations among the variables of interest. To further investigate the hypothesized moderated mediation model, a series of analyses were performed using the SPSS PROCESS macro (Hayes, 2013).

First, the mediating role of self-esteem in the relationship between perceived discrimination and cyberbullying was examined using the PROCESS macro (Model 4) and bootstrapping approaches (Hayes and Preacher, 2014). This model was chosen because it directly evaluates the indirect effect of the perceived discrimination on the cyberbullying via the self-esteem. The indirect relationship between perceived discrimination and cyberbullying through self-esteem was considered significant if the bootstrapped 95% confidence intervals (boot 95% CIs) did not include zero (Aiken et al., 1991).

Second, the moderated mediation effect was examined using the PROCESS macro (Model 15). This model was selected because it allows for the investigation of whether the mediation effect was conditional on a moderator. Demographic variables (age, gender, health, SSES) at T2 were controlled in the analysis. The conditional indirect effect analysis to examine whether the direct and indirect effect of perceived discrimination on cyberbullying significantly differed at high (mean + 1 standard deviation) and low (mean - 1 standard deviation) levels of each moderator (CS, RUS, SCS). Additionally, a simple slope analysis was conducted to explore the nature of the moderation models.

## Results

### Bivariate correlation analysis

The descriptive information and bivariate correlations among the key variables are presented in Table 1. Perceived discrimination (PD) at T1 was positively associated with cyberbullying (CB) at T2 and negatively associated with self-esteem (S-E), CS, RUS, and SC at T2. The result suggests that individuals who perceive higher levels of discrimination are more likely to experience threats to their self-esteem and engage in cyberbullying.

**Table 1:** Descriptive statistics and bivariate correlation analysis.

Variable	Mean	SD	PD (T1)	RUS (T2)	CS (T2)	SC (T2)	S-E (T2)	CB (T2)
PD (T1)	2.545	0.754	—					
RUS (T1)	2.935	0.539	−0.498^***^	—				
CS (T1)	3.464	0.495	−0.140^***^	0.017	—			
SC (T1)	3.199	0.369	−0.458^***^	0.742^***^	0.683^***^	—		
S-E (T2)	2.888	0.453	−0.331^***^	0.424^***^	0.339^***^	0.514^***^	—	
CB (T2)	1.073	0.260	0.085^*^	−0.098^**^	−0.028	−0.086^*^	−0.118^**^	—

*p < 0.05, **p < 0.01, ***p < 0.001; T1, Time 1; T2, Time 2; PD, perceived discrimination; S-E, self-esteem; CS, compassionate self-response; RUS, reduced negative self-responding; SC, self-compassion; CB, cyberbullying.

Moreover, CB at T2 was negatively associated with S-E, CS, RUS, and SC at T2, while was not significantly associated with age and SSES. The finding indicates that higher levels of cyberbullying behaviors were associated with lower self-esteem and lower self-compassion. Additionally, the results of Spearman correlation analysis showed that CB was positively associated with gender (*r* = 1.115, *p* < 0.001; male = 1). The finding indicates that males were more likely to engage in cyberbullying than females.

### Mediation analysis

Table 2 presents the results of the mediation analysis. After controlling for age, gender, health, and SSES, the results of Model 1 showed that PD at T1 negatively predicted self-esteem at T2 (B = −0.173, SE = 0.020, Boot 95% CI [−0.212, −0.135]), and the results of Model 2 showed that S-E at T2 negatively predicted CB at T2 (B = −0.061, SE = 0.020, Boot 95% CI [−0.100, −0.021]). The findings indicated that the total effect of PD at T1 on CB at T2 was significant (total effect = 0.028, SE = 0.001, Boot 95% CI [0.004, 0.051]). Moreover, the indirect effect of PD at T1 on CB at T2 through S-E at T2 was significant (indirect effect = 0.009, SE = 0.003, Boot 95% CI [0.003, 0.019]), although the direct effect of PD at T1 on CB at T2 was non-significant (direct effect = 0.017, SE = 0.012, 95% CI [−0.007, 0.041]).

**Table 2:** Multiple regression analysis.

	Model 1			Model 2		
	Outcome: S-E (T2)			Outcome: CB (T2)		
	*B*	Boot SE	Boot 95% CI	*B*	Boot SE	Boot 95% CI
Gender	0.027	0.028	[−0.029, 0.082]	0.067^***^	0.017	[0.033, 0.101]
Age	0.011	0.010	[−0.008, 0.031]	0.005	0.006	[−0.007, 0.017]
SSES	0.078^***^	0.012	[0.054, 0.102]	0.002	0.008	[−0.013, 0.017]
PD (T1)	−0.173^***^	0.020	[−0.212, −0.135]	0.017	0.012	[−0.007, 0.041]
S-E (T2)				−0.061^**^	0.020	[−0.100, −0.021]
*F*	39.174^***^			6.444^***^		
*R* ^2^	0.150			0.035		

Total effect = 0.028, SE = 0.01, 95% CI [0.004, 0.051]. Direct effect = 0.017, SE = 0.012, 95% CI [−0.007, 0.041]. Indirect effect = 0.010, SE = 0.004, Boot 95% CI [0.003, 0.019]. *p < 0.05, **p < 0.01, ***p < 0.001; number of bootstrap samples = 5,000; SSES, subjective socioeconomic status; PD, perceived discrimination; S-E, self-esteem; CB, cyberbullying.

### Test of moderated mediation model

The PROCESS macro (Model 15) was used to examine the hypothesized moderated mediation model, and the results are shown in Table 3. First, the interaction between RUS at T1 and PD at T1 had a significant effect on CB at T2 (B = −0.045, SE = 0.019, 95% CI [−0.082, −0.008]), indicating that RUS at T1 significantly moderated the direct association between PD at T1 and CB at T2. Moreover, the interaction between RUS at T1 and S-E at T2 had a significant effect on CB at T2 (B = −0.081, SE = 0.033, 95% CI [−0.146, −0.015]), indicating that RUS at T1 significantly moderated the indirect association between PD at T1 and CB at T2 through S-E at T2. According to the result of the conditional direct effect analysis, compared with individuals with low levels of RUS at T1 (i.e., the level of RUS at *M* - 1SD), the indirect effect of PD at T1 on CB at T2 through S-E at T2 was weaker among individuals with high levels of RUS at T1 (i.e., the level of RUS at *M* + 1SD). The result of simple slope analysis is shown in Figure 1.

**Table 3:** Moderated mediation model.

	Outcome: CB (T2)		
	*B*	SE	95% CI
Model 3			
PD (T1)	0.008	0.014	[−0.019, 0.035]
RUS (T1)	−0.023	0.020	[−0.062, 0.016]
S-E (T2)	−0.051^*^	0.021	[−0.092, −0.010]
RUS (T1) × PD (T1)	−0.045^*^	0.019	[−0.082, −0.008]
RUS (T1) × S-E (T2)	−0.081^*^	0.033	[−0.146, −0.015]
Model 4			
PD (T1)	0.021	0.013	[−0.003, 0.046]
CS (T1)	0.006	0.019	[−0.031, 0.043]
S-E (T2)	−0.058^**^	0.021	[−0.099, −0.017]
CS (T1) × PD (T1)	−0.011	0.021	[−0.052, 0.030]
CS (T1) × S-E (T2)	−0.027	0.035	[−0.096, 0.043]
Model 5			
PD (T1)	0.017	0.013	[−0.009, 0.043]
SC (T1)	−0.017	0.028	[−0.075, 0.036]
S-E (T2)	−0.051^*^	0.022	[−0.095, −0.008]
SC (T1) × PD (T1)	−0.076^*^	0.031	[−0.137, −0.014]
SC (T1) × S-E (T2)	−0.103^*^	0.044	[−0.189, −0.017]

*p < 0.05, **p < 0.01, ***p < 0.001; T1, Time 1; T2, Time 2; PD, perceived discrimination; S-E, self-esteem; CS, compassionate self-response; RUS, reduced negative self-responding; SC, self-compassion; CB, cyberbullying.

**Figure 2:**
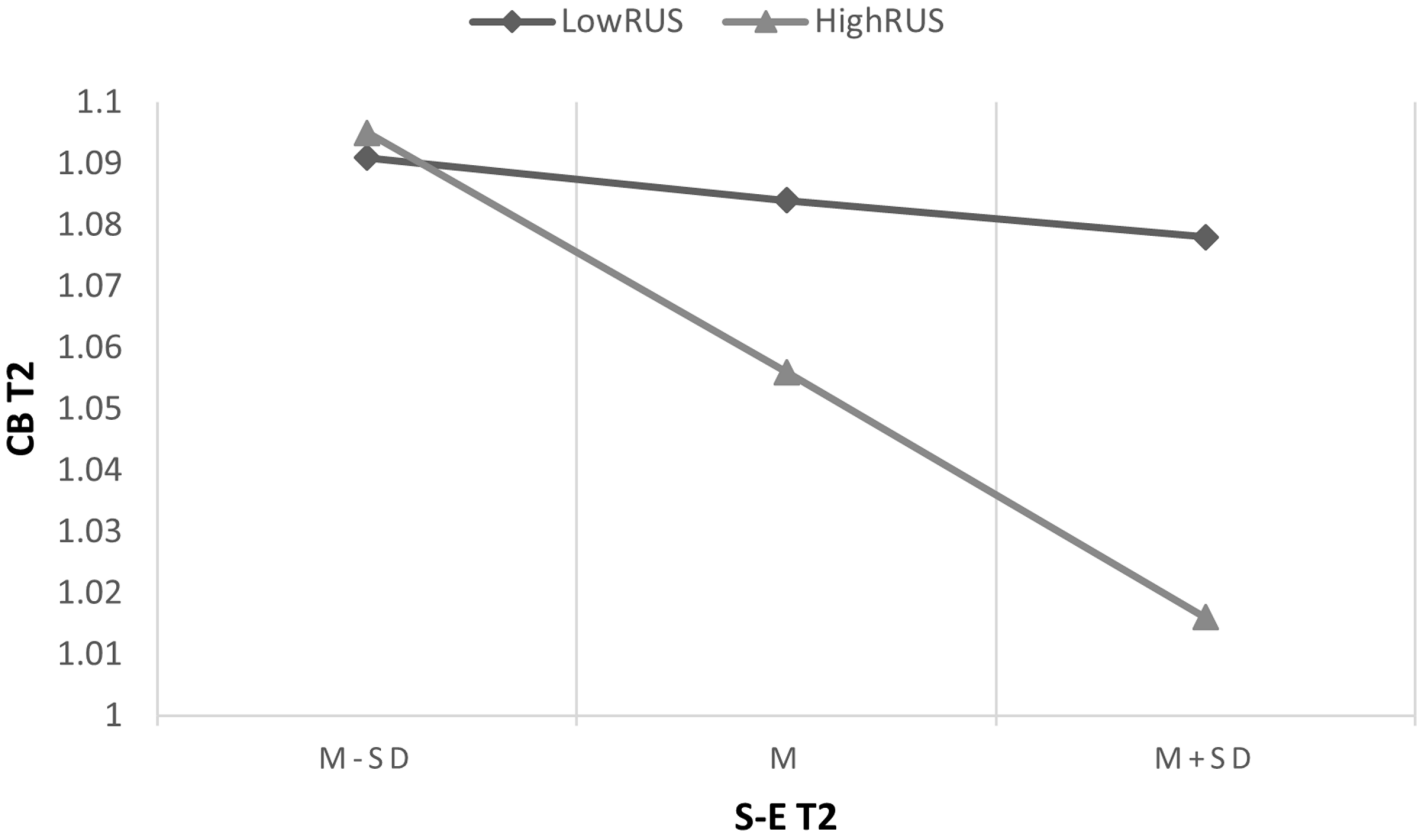
Simple slope analysis. T2, Time 2; RUS, reduced uncompassionate self-responding; CB, cyberbullying; S-E, self-esteem.

**Figure 3:**
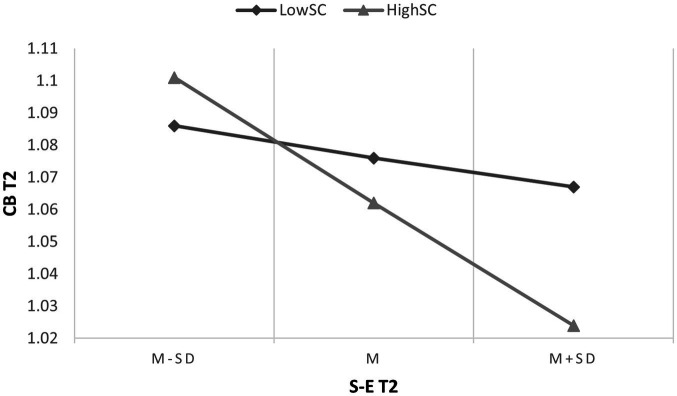
Simple slope analysis for overall self-compassion. T2, Time 2; CB, cyberbullying; SC, self-compassion; S-E, self-esteem.

Second, the results of Model 5 are shown in Table 3. The interaction between SC at T1 and PD at T1 had a significant effect on CB at T2 (B = −0.076, SE = 0.031, 95% CI [−0.137, −0.014]), indicating that SC at T1 significantly moderated the direct association between PD at T1 and CB at T2. Moreover, the interaction between SC at T1 and S-E at T2 had a significant effect on CB at T2 (B = −0.103, SE = 0.044, 95% CI [−0.189, −0.017]), indicating that SC at T1 significantly moderated the indirect association between PD at T1 and CB at T2 through S-E at T2. According to the results of the conditional direct effect analysis, compared with individuals with low levels of SC at T1 (i.e., the level of SC at *M* - 1SD), the indirect effect of PD at T1 on CB at T2 through S-E at T2 was weaker among individuals with high levels of SC at T1 (i.e., the level of SC at *M* + 1SD). The simple slope analysis is shown in Figures 2, 3. Additionally, the results of Model 4 indicated that the moderating effect of CS at T1 on the direct and indirect association between PD at T1 and CB at T2 through decreased S-E was non-significant (Table 3).

## Discussion

The purpose of this study was to explore how perceived discrimination is related to cyberbullying through self-esteem and test the moderating roles of compassionate self-response, reducing uncompassionate responses, and overall self-compassion in reducing cyberbullying. This aligns with the stress coping theory (Lazarus, 1993). Cyberbullying has been previously identified as a negative coping behavior in response to stressful events (e.g., perceived discrimination) (Wang et al., 2022), and the same pattern was observed in our study among college students. The findings highlight that perceived discrimination can lead to decreased self-esteem, which in turn increases cyberbullying among college students.

The findings indicate that the relationship between perceived discrimination and cyberbullying is mediated by self-esteem, which highlights the role of global self-esteem in this dynamic. Self-esteem is understood as an individual’s subjective evaluation of their relationships with society and significant others, reflecting their status in interpersonal relations. Individuals with low self-esteem may internalize feelings of rejection from their experiences of frustration, which can lead to aggressive behaviors such as cyber aggression (Barry et al., 2007). Specifically, when individuals face criticism, rejection, exclusion, or discrimination from others they may experience feelings of inferiority and shame, and these feelings can trigger anger and hostility towards others, potentially leading to cyberbullying (Shi et al., 2017). Moreover, the theory of threatened egotism (Baumeister et al., 1996) posits that individuals with fragile self-esteem are particularly vulnerable to threats to their self-worth, such as perceived discrimination. When their self-esteem is challenged, they may experience a heightened sense of threat to their social identity, leading to defensive behaviors. Low self-esteem heightens psychological vulnerability, shaping individuals’ maladaptive coping strategies. As a maladaptive response, some students may engage in cyberbullying to regain a sense of control in online interactions. The anonymity and reduced accountability of digital spaces can further contribute to such behaviors. Accordingly, addressing self-esteem as a mediating factor is crucial in interventions targeting cyberbullying reduction.

Moreover, this study is the first to explore the buffering role of different dimensions of self-compassion in understanding the indirect association between perceived discrimination and cyberbullying. The findings reveal that uncompassionate self-responding can weaken the direct association of between perceived discrimination and cyberbullying and its indirect association through self-esteem. According to stress coping theory, individuals with low RUS levels are more likely to engage in cyberbullying. Previous studies have found that higher self-compassion is associated with greater use of adaptive coping strategies and less use of maladaptive coping strategies in stressful situations, such as discrimination (Ewert et al., 2021). This implies that college students with high RUS are more likely to use adaptive coping strategies rather than resorting to cyberbullying to manage stressful events.

Furthermore, individuals with high level of RUS are more likely to reduce the negative effects of a damaged self-esteem and less likely to engage in cyberbullying when they experience high levels of discrimination. The findings highlight the importance of developing self-compassion as a potent psychological resource for the prevention and reduction of cyberbullying behavior, particularly for individuals facing frustration. This study did not find any moderating effect of CS on the examined pathways. Empirical studies have indicated that CS is more strongly associated with the disappearance of internalizing symptoms (Li et al., 2022; Muris et al., 2021) whereas uncompassionate self-responding is more likely to be related to external behavior, such as behavior disorders (Bicaker and Racine, 2022). These findings suggest that CS is more effective in reducing internalizing symptoms rather than behaviors that involve outward aggression, such as cyberbullying. This might explain the minimal correlation between CS and the problematic behavior of cyberbullying found in the present study.

The moderating effect of RUS on the relationship between perceived discrimination and cyberbullying is a critical finding in understanding how individuals can manage negative emotional responses to discrimination. This distinction between CS and RUS highlights the importance of considering different aspects of self-compassion in interventions aimed at reducing cyberbullying. The finding implicates that RUS may serve as a more relevant resource for managing the negative effects of perceived discrimination, particularly in situations where individuals are at risk of engaging in harmful external behaviors. RUS refers to the reduction of harsh, self-critical, and uncompassionate thoughts and behaviors, fostering a more balanced and compassionate self-view (Neff et al., 2018). Specifically, individuals with high RUS are more likely perceived less the negative emotional consequences of perceived discrimination, such as feelings of shame, anger, and frustration, which can further reduce the risk of engaging in aggressive behaviors like cyberbullying. By cultivating a more compassionate and less judgmental response to their own emotional pain, individuals can reduce the likelihood of externalizing these emotions in aggressive ways.

A number of limitations of the present study should be considered. First, data were collected from only two waves to examine the longitudinal mediation mechanism, which may lead to biased results. Future research should consider collecting data across three or more waves to examine the temporal stability of these relationships and to explore how changes in self-esteem over time may influence the occurrence of cyberbullying. Second, the data were collected from Chinese university students, which may limit the generalizability of the findings. Thus, future studies should aim to include more diverse populations, such as students from different countries, adolescents, or individuals from varying socioeconomic backgrounds, to examine whether these findings hold across different contexts.

In summary, this study highlights the mediating role of impaired self-esteem in the pathway from perceived discrimination to cyberbullying behavior, and examines the role of RUS in mitigating impaired self-esteem to reduce cyberbullying in the context of discrimination experiences. This research provides new insights for the development of interventions to reduce cyberbullying and maintain a civilized online environment. Given the pivotal role of reduced uncompassionate self-judgment (RUS) in this process, targeted interventions, such as cognitive-behavioral techniques and RUS-focused mental health programs, could effectively prevent cyberbullying, especially among students experiencing perceived discrimination.

## Data Availability

The raw data supporting the conclusions of this article will be made available by the authors, without undue reservation.
